# Fabrication of Electrospun Ni_0.5_Zn_0.5_Fe_2_O_4_ Nanofibers Using Polyvinyl Pyrrolidone Precursors and Electromagnetic Wave Absorption Performance Improvement

**DOI:** 10.3390/polym13234247

**Published:** 2021-12-03

**Authors:** Kyeong-Han Na, Kyong-Pil Jang, Sung-Wook Kim, Won-Youl Choi

**Affiliations:** 1 Department of Advanced Materials Engineering, Gangneung-Wonju National University, Gangneung 25457, Korea; nag0717@naver.com; 2 Korea Institute of Civil Engineering and Building Technology, Goyang 10223, Korea; kyongpiljang@kict.re.kr (K.-P.J.); swkim@kict.re.kr (S.-W.K.); 3 Research Institute for Dental Engineering, Gangneung-Wonju National University, Gangneung 25457, Korea

**Keywords:** microwave absorber, electrospinning, nanofibers, NiZn ferrite, return loss

## Abstract

Ni_0.5_Zn_0.5_Fe_2_O_4_ nanofibers with an average diameter of 133.56 ± 12.73 nm were fabricated by electrospinning and calcination. According to our thermogravimetric—differential thermal analysis and X-ray diffraction results, the calcination temperature was 650 °C. The microstructure, crystal structure, and chemical composition of the nanofibers were observed using field-emission scanning electron, X-ray diffraction, and energy-dispersive X-ray spectroscopy. Commercial particle samples and samples containing 10 wt% and 20 wt% nanofibers were fabricated, and the electromagnetic properties were analyzed with a vector network analyzer and a 7.00 mm coaxial waveguide. Regardless of the nanofiber content, Ni_0.5_Zn_0.5_Fe_2_O_4_ was dominantly affected by the magnetic loss mechanism. Calculation of the return loss based on the transmission line theory confirmed that the electromagnetic wave return loss was improved up to −59.66 dB at 2.75 GHz as the nanofiber content increased. The absorber of mixed compositions with Ni_0.5_Zn_0.5_Fe_2_O_4_ nanofibers showed better microwave absorption performance. It will be able to enhance the performance of commercial electromagnetic wave absorbers of various types such as paints and panels.

## 1. Introduction

With the development of wireless communication technology, the use of portable electronic devices has rapidly increased. At the same time, there are many concerns about the harmful effects of electromagnetic (EM) waves on the human body [1,2,3], and EM waves other than the used frequency decrease the performance of a device, so the demand for EM wave shielding is also increasing. Using metal as a conductive shielding material suppresses interference via reflecting incident EM waves to protect internal circuits and the human body. However, a metal EM shield is heavy and expensive to maintain, and noise generated inside might create reflecting incident EM waves in the circuit. An electromagnetic wave absorber has been proposed as an alternative solution. The absorbing materials convert incident EM waves into thermal energy and dissipate them through something like dielectric and magnetic mechanisms. The absorber can be applied in the form of tiles, films, paints, etc. The dielectric and magnetic properties of filler determine the absorbing performance. Therefore, it is important to select an absorber that has suitable EM properties for the required absorbing performance and used frequency. Materials such as carbon materials [4,5,6], carbonyl iron [7,8,9], and various ferrite materials [10,11,12] are mainly studied as absorbers because they are light, thin, and small but have a superior EM wave absorption performance. As for a magnetic absorber, soft magnetic spinel ferrite has mainly been studied and shown to modify the EM wave performance by substituting one or more divalent metal ions like Mn [13,14,15], Co [16,17,18], Ni [19,20,21], Cu [22,23,24], and Mg [25,26] at the site of Fe atoms.

At the same time, the morphological character of the absorbent particles has a great influence on the EM wave absorbing performance and the selection of frequency. Even if absorbers have the same chemical composition, they need to be controlled the microstructure. Many studies have been carried out to modify or optimize the absorber by applying various microstructures: mesoporous structures [27,28], core–shell structures [29,30], nanoflakes [31,32], nanofibers [33,34], etc. The nanostructured particles have superior properties such as a large specific surface area and many dangling bonds of surface atoms, and they exhibit excellent microwave absorption characteristics due to interfacial polarization and multiple scattering. Much research has been conducted to improve performance by manipulating the shape of the particles; the structures studied mainly included sphere, rhombus, arborization, flake, acicular, and fibrous structures. The shape of the absorbent has an immediate influence on the EM parameters and scattering effect, and it is considered that the performance is improved when the absorbents contain the anisotropy structure. There are many methods to synthesize various nanostructures: hydrothermal [35], precipitation-thermal decomposition [28], electrospinning [33], metal–organic chemical vapor deposition [36], template [37], and sol-gel method [38].

Among them, electrospinning is a method to fabricate nanofibers by applying a high voltage to two spaced electrodes and jet spraying and stretching a precursor solution using the generated electrostatic force. Due to stretching, the solvent is volatilized with a rapid increase of surface area and the solute remains in the form of very thin fibers. As a result, a one-dimensional structure with a high aspect ratio and a nanoscale diameter can be obtained, and it can be transformed to ceramic nanofibers via calcination. Since it is a continuous process that can obtain very long and uniform nanofibers, it is being applied in various fields such as biomedical [39], photovoltaic [40], and optics [41].

In this study, we fabricated Ni_0.5_Zn_0.5_Fe_2_O_4_ (NZF), which is often discussed as an EM wave absorber [42] as a nanofiber structure via the electrospinning process. To improve enhanced absorption performance through chemical composition and microstructure control, the NZF composition that is very attractive in EM absorber was selected and fabricated to nanofiber structure having higher shape magnetic anisotropy. Compared to the conventional absorber with isotropic ferrite powder, the absorber of mixed compositions with anisotropic NZF nanofibers can expect better electromagnetic wave absorption performance. The NZF nanofibers were fabricated by electrospinning and calcination. Precursor solutions that can be used for electrospinning were prepared, and calcination conditions for as-spun nanofibers were determined using thermogravimetric analysis and X-ray diffraction. The microstructure and diameter of the calcined nanofibers were confirmed using FE-SEM. We prepared samples for measuring the EM properties by mixing the electrospun nanofibers with commercially available nanopowders and calculated the reflection loss for each thickness using transmission line theory. We measured the EM properties by a vector network analyzer (VNA).

## 2. Materials and Methods

### 2.1. Chemicals

The reagents used to prepare the precursor solutions were as follows: Ni(NO_3_)_2_·6H_2_O (EP, Samchun Chemicals Co., Ltd., Seoul, Korea), Zn(NO_3_)_2_·6H_2_O (EP, Daejung Chemicals Co., Ltd., Gyeonggi, Korea), Fe(NO_3_)_2_·9H_2_O (GR, Kanto Chemical Co. Inc., Tokyo, Japan), Polyvinyl pyrrolidone (PVP, M.W. 1,300,000, Alfa Aesar Korea Co., Ltd., Incheon, Korea), and *N*,*N*-dimethylmethanamide (DMF, EP, Daejung Chemicals Co., Ltd., Gyeonggi, Korea), commercial NZF nanoparticles (≥99.5%, SAT nano technology material Co., Ltd., Dongguan, China), epoxy binder (YD-014, Kukdo Chemical, Seoul, Korea).

### 2.2. Electrospinning Process

**Figure 1:**
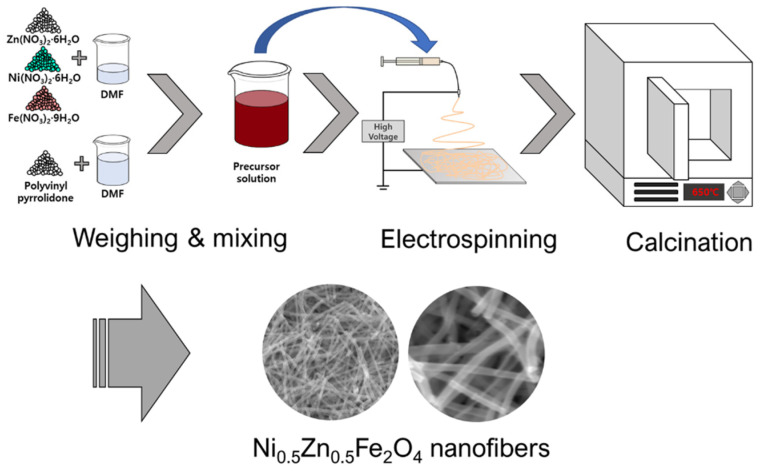
Schematic diagram of the fabrication process with electrospinning and calcination.

To fabricate NZF nanofibers via electrospinning, we prepared a precursor solution. First, 10.0 g of PVP was mixed with 60.0 g of DMF for 6 h using a magnetic stirrer. In another beaker, 40 mmol of Fe(NO_3_)_2_·9H_2_O, 10 mmol of Ni(NO_3_)_2_·6H_2_O, and 10 mmol of Zn(NO_3_)_2_·6H_2_O were added to 30.0 g of DMF and mixed for 2 h. Then, the second solution was added to the first solution and stirred for 6 h. The prepared precursor solution was loaded into a 12 mL polypropylene syringe with an inner diameter of 15.56 mm. The syringe was connected with a stainless-steel nozzle adapter and a 23 gage capillary using polypropylene tubing. The distance between the tip of the capillary and the grounded collector was set up to 20 cm. Then the adapter and collector were connected to the power supply and a high voltage of 23 kV was applied to start the electrospinning process. A flow rate of 0.2 mL per hour was constantly applied to the syringe using a syringe pump. Room conditions were temperature of 24 °C and humidity less than 40%. As spun nanofibers were physically separated from the collector using a Teflon tweezer and then dried at 70 °C for 6 h in a dry oven. After that, dried nanofibers were calcined using a box furnace. The calcination conditions were as follows: stabilized at 30 °C, ramping to 200 °C, hold 2 h at 200 °C, ramping to 650 °C and hold 2 h. The temperature ramping speed was 5 °C per minute. The schematic diagram of the fabrication process is shown in Figure 1.

### 2.3. Fabrication of RL Measuring Sample

Since calcined nanofibers are obtained as scaffolds, a dispersing process was needed. Nanofibers were added to 75% ethanol in a 50 mL vial and sonicated for 2 h and dried at 70 °C in a dry oven. The calcined nanofibers/commercial particle NZF mixture was mixed for each other content; 0 wt%, 10 wt%, and 20 wt% of nanofibers/nanoparticles powder of 0.45 g and epoxy binder of 0.05 g were mixed using a mortar. The prepared powders were uniaxially pressed to fabricate toroidal samples (D_out_ = 7.00 mm, D_in_ = 3.03 mm) under the pressure of 250 MPa and then were cured at 200 °C for 30 min.

### 2.4. Characterization

To determine the calcination temperature, thermogravimetric—differential thermal analysis (TGA-DTA) of the as-spun nanofibers was carried out using a Thermogravimetric Analyzer (TGA, STA 409, NETZSCH, Hanau, Germany). The crystal structure of the calcined nanofibers calcined at each different temperature was analyzed by an X-ray diffractometer (XRD, AXS-D8, Bruker, Billerica, MA, USA). The morphology and microstructure of the NZF nanofibers and commercial particles were analyzed by a field emission scanning electron microscope (FE-SEM, Inspect F, FEI Korea Co., Ltd., Gyeonggi, Korea) and energy-dispersive X-ray spectroscopy (EDS). To obtain the complex permeability and permittivity, an electromagnetic property test of the prepared toroidal samples was carried out using a vector network analyzer (VNA, N5222B, Keysight, Santa Rosa, CA, USA).

## 3. Results and Discussion

**Figure 2:**
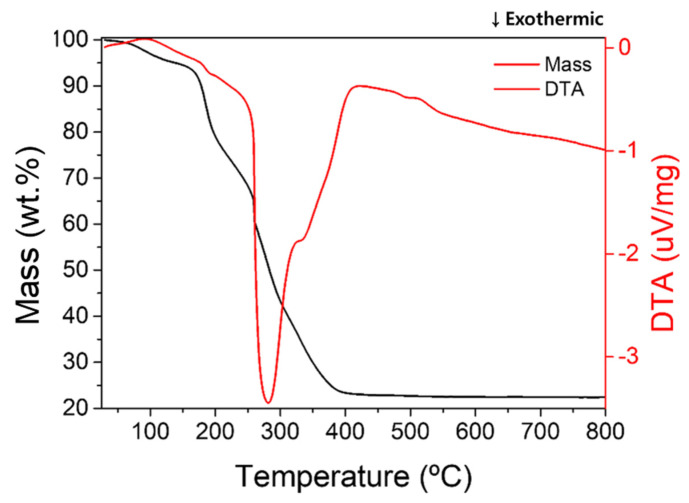
TGA curve of as-spun NZF nanofibers.

The TGA-DTA was carried out to determine the calcination temperature of the as-spun nanofibers and the result is presented in Figure 2.

An initial mass decrease of about 6% occurred in the temperature range of 60 °C to 140 °C, and it was likely due to the volatilization of adsorbed moisture and residual solvent. The DTA peaks are 100 °C and 135 °C, respectively, and these reactions are endothermic near the boiling points of water and DMF. Endothermic DTA peaks are observed at 100 °C, 135 °C and it is near the boiling point of water and DMF. The mass loss of 16%, starting at around 180 °C, lasts up to 200 °C. The mass-loss rate decreased from 200 °C to 250 °C and then increased. This result explained how the endothermic reaction of glass transition suppressed the exothermic of PVP decomposition. After that, a strong exothermic reaction and mass loss were observed near 260 °C, which was likely rapid PVP decomposition by ignition and combustion. Then there was a continuous exothermic reaction and mass loss by the decomposition of residual carbon black and crystallization of metal ion.

**Figure 3:**
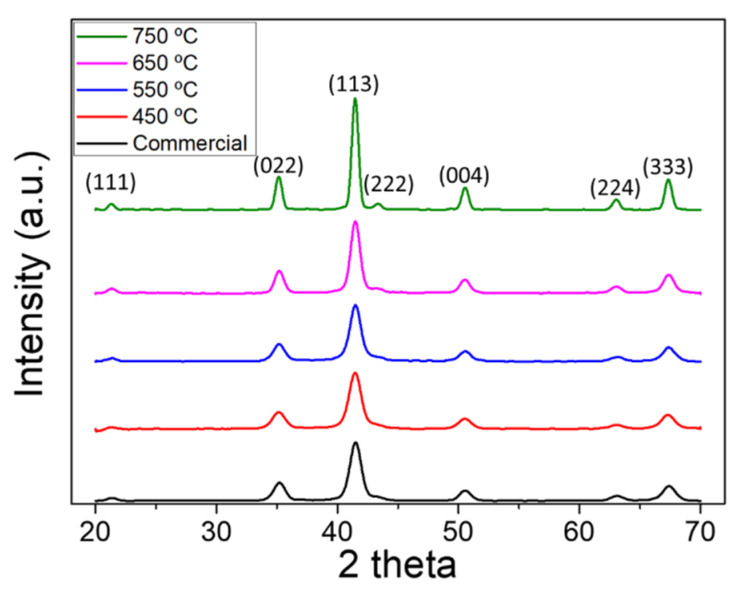
X-ray diffraction patterns of the commercial NZF nanoparticles and electrospun NZF nanofibers calcined at 450 °C, 550 °C, 650 °C, and 750 °C.

Figure 3 shows the XRD patterns of commercial NZF nanoparticles and NZF nanofibers calcined at various temperatures. According to the TGA result, all volatile components were removed, and only inorganic components remained at a temperature above 450 °C; the XRD pattern was also confirmed as a crystallized phase. The Ni_0.5_Zn_0.5_Fe_2_O_4_ spinel structure was confirmed by comparing it with the Crystallography Open Database ID 96-900-9921 [43], and the Miller index of each significant peak was labeled. From 450 °C to 750 °C, the crystal structure did not change and the peak gradually sharpened, which means the crystallinity of nanofibers was increasing. To confirm more accurately, the crystallite size of each sample was calculated via the Scherrer equation ($D_{p}=0.94\lambda /\beta \cos\theta$), and the results are shown in Table 1, where $D_{p}$ is the represent average crystallite size, β = the full width at half maximum (FWHM) of the peak, θ = the Bragg angle, and λ is the wavelength of the X-ray used for diffraction (K_α1_ = 1.78897 Å).

As expected from the pattern, the average crystallite size gradually increased according to calcination temperature, and rapid growth was observed at 750 °C. Since this study focused on the geometrical advantages of electrospun nanofibers as an EM wave absorber, the calcination temperature was determined as 650 °C, which has the most similar crystallite size to commercial nanoparticles. Considering that the shape factor compared to nanoparticles is reduced due to the shape anisotropy of the 1-D nanostructure, 650 °C was selected not 550 °C.

**Figure 4:**
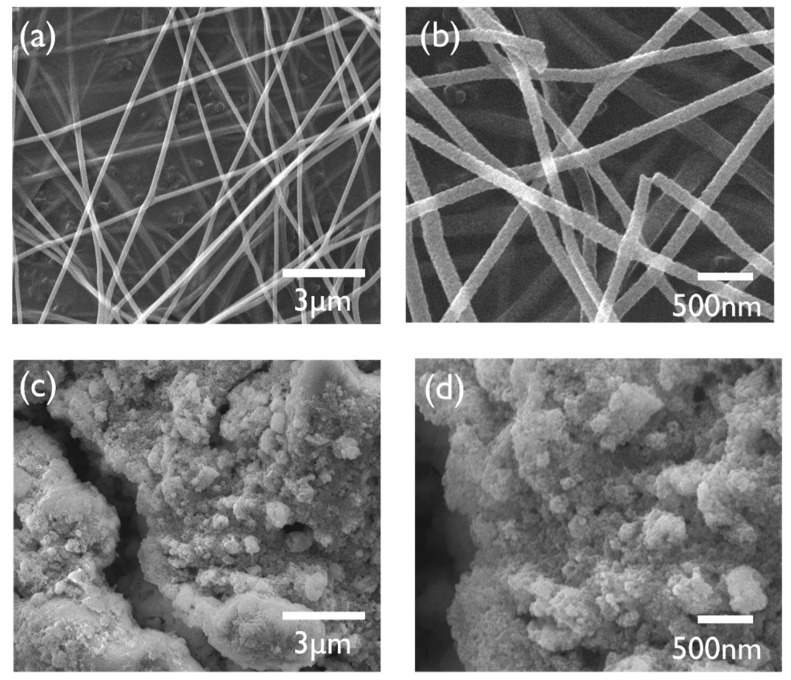
FE-SEM images of calcined NZF nanofibers obtained by electrospinning: (**a**) low magnification of the nanofibers, (**b**) high magnification of the nanofibers, (**c**) low magnification of the commercial nanoparticles, and (**d**) high magnification of the commercial nanoparticles.

Figure 4 shows the FE-SEM image of electrospun NZF nanofibers and commercial nanoparticles. These images demonstrate that the proposed process conditions of electrospinning and calcination are appropriate for the fabrication of a 1-D nanostructure. Nanofibers with a smooth surface were fabricated, and untargeted structures due to unoptimized process conditions such as bead structure, particle continuum due to over crystallization were not confirmed. The average diameter of the nanofibers was 133.56 ± 12.73 nm. The chemical composition of the nanofibers was analyzed using EDS and the results of the quantitative analysis are shown in Table 2. The atomic ratio of each element was close to being consistent with the target composition, Ni: Zn: Fe: O = 1:1:4:8. Commercial nanoparticles were observed as simple particles with no specific structure.

**Figure 5:**
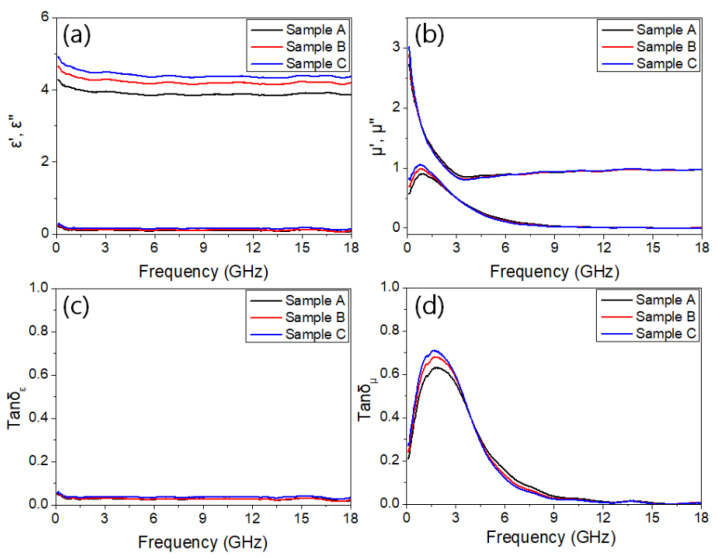
EM properties of NZN composite samples: (**a**) real and imaginary permittivity, (**b**) real and imaginary permeability, (**c**) electric loss tangent, and (**d**) magnetic loss tangent.

The electromagnetic properties could be explained using permittivity and permeability. These material constants were expressed as a complex form, namely, ε_r_ = ε′ − jε″ and µ_r_ = µ′ − jµ″. The real parts are related to the storage of electromagnetic energy and the imaginary parts show the dissipation of energy. Each real and imaginary part of the complex permittivity and permeability of the NZF nanofibers is shown in Figure 5a,b.

These constants were obtained using VNA and a 7.00 mm coaxial waveguide. Samples A, B, and C contained 0 wt%, 10 wt%, and 20 wt% of NZF nanofibers, respectively. The composition of each sample is shown in Table 3.

There was a tendency for the coefficient to according to the content of nanofibers. The permittivity of the samples was almost independent of frequency. The real parts of the permittivity were measured from 4.01 to 4.91 and imaginary parts were almost near 0, which means that the permittivity of the NZF absorbent has almost no influence on a change in the EM wave absorbing performance. At the same time, real parts of permeability showed a decreasing tendency from 2.72 to 0.85 at the ~3.56 GHz frequency. The peaks of imaginary parts were observed to be near 1 at 0.79 GHz. All the permeability coefficients decreased with an increasing frequency, which was likely due to magnetization relaxation. The ratio of the imaginary part related to energy dissipation and the real part showed energy storage called electric and magnetic loss tangent, which are written as tan *δ*_ε_ = *ε*″/*ε*′, tan *δ*_µ_ = *µ*″/*µ*′, respectively. In general, it is expected that the larger the loss tangent and imaginary part of the absorber, the better the EM wave absorbing performance. The dielectric and magnetic loss tangents are shown in (c) and (d) of Figure 5. While the electrical loss tangent was close to 0 at all frequency ranges, the maximum value of the magnetic loss tangent was 0.69, which confirmed that the EM wave performance of NZF largely depends on the magnetic loss. Magnetic losses are usually due to resonance, eddy currents, and hysteresis loss. Figure 5d shows that samples B, and C had larger *μ*′ and *μ*″ than sample A due to the high shape magnetic anisotropy of the nanofiber structure. Multiple magnetic resonance loss was not observed, and the magnetic wall resonance in the low-frequency range is dominant. High shape magnetic anisotropy of nanofibers makes it difficult to change the magnetic domain direction and increase the demagnetizing energy, so hysteresis loss which is mainly contributed by changing the domain direction is increased. Since the microstructures containing nanofibers have higher porosity than those without, samples B and C are expected to have a larger surface area than sample A. Therefore, it can be expected that increasing magnetic loss via eddy current. Since eddy current loss is defined by permeability, thickness, and electrical conductivity, the better magnetic loss can be expected from Sample C, which has the largest imaginary part of complex permeability. The eddy current loss factor can be evaluated according to the following Equation (1) [44,45].

(1) $$C_{0}=\mu^{\prime \prime }{\left(\mu^{\prime }\right)}^{-2}f^{-1}=2\pi \mu_{0}d^{2}\sigma$$

In this formula, $C_{0}$ is eddy current loss coefficient, *f* is the frequency, *d* is thickness, and σ is the electrical conductivity. We measured the complex permittivity and complex permeability for each frequency, so the return loss (RL) value with the thickness of the absorber could be calculated by the following Equations (2)–(4) based on the transmission lint theory:

(2) $$Z_{in}=Z_{0}\sqrt{\left(\frac{\mu_{r}}{\varepsilon_{r}}\right)}tanh\left[\left(j\frac{2\pi fd}{c}\right)\right)\sqrt{\mu_{r}\cdot \varepsilon_{r}}]$$

(3) $$Z_{0}=\sqrt{\left(\frac{\mu_{0}}{\varepsilon_{0}}\right)}$$

(4) $$RL\left(dB\right)=20\;log\left|\frac{Z_{in}+Z_{0}}{Z_{in}-Z_{0}}\right|$$

where $Z_{0}=377\Omega$ is the characteristic impedance of free space, $\mu_{r}$ and $\varepsilon_{r}$ represent the complex permittivity and permeability of the materials, *d* is thickness of the absorber layer, and *c* is the velocity of the EM wave in free space. By substituting the obtained variables into (2), the incident impedance *Z_in_* of the absorber is calculated, and the return loss can be obtained by substituting *Z_in_* and the free space impedance *Z*_0_ into (4).

**Figure 6:**
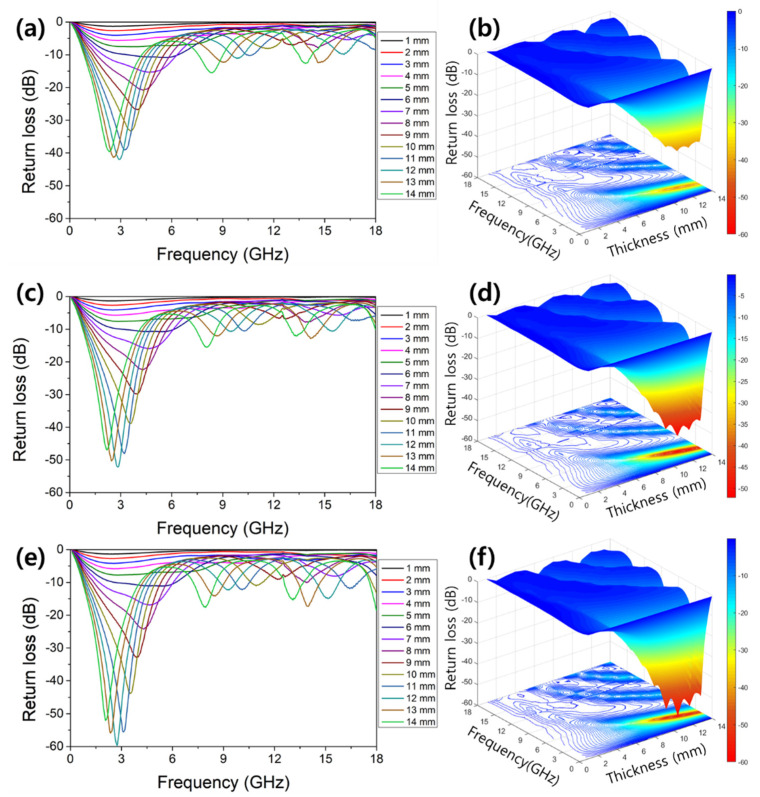
Frequency and thickness dependences of the reflection loss of NZF: (**a**,**b**) reflection loss graphs, 3D map, and contour of sample A, (**c**,**d**) reflection loss graphs, 3D map, and contour of sample B, (**e**,**f**) reflection loss graphs, 3D map, and contour of sample C.

Figure 6 shows the results of the calculated RL value for each thickness and frequency using the above impedance and return loss equation. (a), (c), and (e) on the left side are graphs showing the RL value according to the thickness and frequency of samples A, B, and C, respectively, and (b), (d), and (f) on the right side are those three-dimensional plots and a contour line. The tendency of the increasing RL value in proportion to the nanofibers content was clearly shown, and the shift of the peak frequency was slight. For all samples, the minimum RL value was confirmed at 12 mm thickness, and at the peak data, the frequency was 2.75~2.89 GHz, and the RL value was −41.99~−59.66 dB. Nanomaterials such as nanoparticle and nanofiber tend to agglomerate due to various reasons such as surface free energy contributed by a large specific surface area, weak electric double layer repulsion, and limitation of mechanical dispersion, so it is difficult to disperse nanomaterials in the matrix. Well-dispersed absorber network is an important issue as it can show optimal performance by making the local impedance uniform, increasing the interface with free space, and having benefits in incident wave absorption and heat dissipation [46,47]. Although this study focused on EM properties and performances of absorbers which have the same chemical composition and the difference microstructure, well-dispersed absorbers of NZF nanoparticle and nanofiber will enhance the EM performances. Additional research on the surfactant and surface modification using functional group and heterogeneous structure with like MXenes [46], graphene [47] is needed.

## 4. Conclusions

In this study, we prepared NZF nanofibers using electrospinning and calcination. The crystal structure and 1-D nanostructure were well-formed, and the average diameter was measured to be 133.56 ± 12.73 nm. We observed that the NZF absorber performance was dominantly affected by magnetic loss regardless of the nanofiber content. We measured the EM properties of samples of pure commercial nanoparticles and samples containing nanofibers of 10 wt% and 20 wt%. We found that as the nanofiber content increased, the EM wave absorption performance improved. In all the samples, the frequency at which the minimum RL value was confirmed was near 2.8 GHz and the thickness was near 12 mm. The increasing of absorbing performance due to nanofiber content is consistent with the results of previous studies that demonstrated that the mixed structure of acicular and particles increased the absorber performance. Our study confirmed that the nanofiber structure is a superior structure for improving the performance of the EM wave absorber and is expected to be effective for any type of magnetic absorber, such as panels, films, and paints.

## Figures and Tables

**Table 1:** The calculated average crystallite size of calcined Ni_0.5_Zn_0.5_Fe_2_O_4_ nanofibers.

Sample	Plane	2θ (°)	FWHM (°)	Calculated Average Grain Size (Diameter, nm)
Commercial	(022)	41.5	1.11	9.22
	(131)	35.22	1.12	
	(004)	67.4	1.24	
450 °C	(022)	41.46	1.22	8.44
	(131)	35.14	1.27	
	(004)	67.3	1.3	
550 °C	(022)	41.48	1.15	8.79
	(131)	35.18	1.23	
	(004)	67.38	1.26	
650 °C	(022)	41.48	1.01	10.17
	(131)	35.2	0.97	
	(004)	67.36	1.17	
750 °C	(022)	41.46	0.66	14.51
	(131)	35.14	0.93	
	(004)	67.34	0.68	

**Table 2:** EDX spectrum analysis results for calcined NZF nanofibers.

Element	Weight Percent (%)	Atomic Percent (%)	Net Int.	Error (%)
Fe	46.58	29.71	1082.70	2.56
Ni	13.23	8.03	221.19	5.08
Zn	16.19	8.82	176.36	5.41
O	24.00	53.44	916.52	7.08

**Table 3:** The Composition of Sample A, B, and C.

Sample	NZF Nanoparticles (g)	NZF Nanofibers (g)	Epoxy Binder (g)
A	0.450	-	0.050
B	0.405	0.045	0.050
C	0.360	0.09	0.050

## Data Availability

Not applicable.
